# Asymmetric relationship of urbanization and CO_2_ emissions in less developed countries

**DOI:** 10.1371/journal.pone.0208388

**Published:** 2018-12-07

**Authors:** Julius Alexander McGee, Richard York

**Affiliations:** 1 Department of Sociology, Portland State University, Portland, Oregon, United States of America; 2 Department of Sociology, University of Oregon, Eugene, Oregon, United States of America; Universitat Jaume I, SPAIN

## Abstract

Understanding the relationship between carbon dioxide (CO_2_) emissions and the urbanization of national populations has been a key concern for environmental scholars for several decades. Although sophisticated modeling techniques have been developed to explore the connection between increases in urban populations and CO_2_ emissions, none has attempted to assess whether declines in urbanization have an effect on emissions that is not symmetrical with that of growth in urbanization. The present study uses panel data on CO_2_ emissions and the percentage of individuals living in urban areas, as well as a variety of other structural factors, for less-developed countries from 1960–2010, to empirically assess whether the effect of growth in urban populations on emissions is symmetrical with the effect of decline. Findings indicate that the effect of growth/decline in urban populations on CO_2_ emissions is asymmetrical, where a decline in urbanization reduces emissions to a much greater degree than urbanization increases emissions. We hypothesize that this is at least in part because deurbanization is connected with disruptions to the production and distribution of goods and services and/or access to electricity and other energy sources. Our finding suggests that not only the absolute level of urbanization of nations matters for emissions, but also how the patterns of migration between rural and urban areas change over time. Future research should be mindful of the processes behind deurbanization when exploring socioeconomic drivers of CO_2_ emissions.

## Introduction

Anthropogenic greenhouse gas emissions, especially carbon dioxide (CO_2_) emissions, are widely recognized as the largest contributors to global climate change. A number of studies have found that increases in the number of individuals living in urban areas is a significant contributor to greenhouse gas emissions [1, 2, 3, 4]. Since 2008, the majority of the world’s population has resided in urban areas [5]. Globally, urban populations are projected to continually grow in the coming decades, and the vast majority of that growth is projected to occur in less developed countries (LDCs) [5]. As such, it is crucial to understand how urban development in LDCs is connected to CO_2_ emissions.

Although scholars have used a variety of sophisticated statistical modeling techniques to understand the relationship between urban development and CO_2_ emissions, most modeling techniques implicitly assume a symmetrical relationship between growth and decline in urban development and CO_2_ emissions. While typically not made explicit in research on socioeconomic drivers CO_2_ emissions, the assumption of symmetry suggests that the effect of growth in urban development on emissions is of the same magnitude, but simply opposite in direction, as the effect of decline in urbanization. However, there are sensible reasons to expect that deurbanization is not simply the reverse of urbanization, so that urban growth and decline have asymmetric effects. Assessing the extent to which asymmetric relationships are common should be a key concern for environmental scholars as well as policy makers, as it has clear, although nuanced, implications regarding the consequences of urban change and the development of environmental degradation [6,7].

Here, we focus on the implications of an asymmetric association between urbanization and CO_2_ emissions in LDCs rather than all nations or developed countries. There is no single, standard definition of how to classify a nation as an LDC or as a “developing” nation. Nonetheless, typically LDCs are contrasted with “developed” nations based on levels of GDP per capita, industrial production, the Human Development Index, and other indicators of levels of prosperity and standard of living. Here we refer to nations classified as “low and middle income” by the World Bank as LDCs and those classified as “high income” as developed countries. In many LDCs, more commonly than in developed countries, urban inhabitants frequently maintain social ties to rural regions and over time return to these areas [8,9], creating a circular pattern of urban to rural migration. During periods of industrial decline, the flow of migration from urban to rural areas increases, reducing CO_2_ from industrial development. Moreover, in LDCs day-to-day living in rural areas is far less fossil fuel intensive than in urban areas due to lack of rural infrastructure, so urban-rural shifts in population can have large effects on CO_2_ emissions. In these circumstances, it is likely that people moving from an urban to a rural area will see an immediate drop in energy use due to lack of access to electricity and other energy sources. However, people moving from a rural to an urban area are unlikely to see an immediate increase in energy use, since they will need time to connect with the urban economy and establish an urban standard of living. For these reasons, we hypothesize that these processes may result in deurbanization in LDCs reducing emissions more than urbanization increases emissions. Our findings have broader implications for policies promoting further social integration of rural and urban regions in LDCs as a pathway toward combating climate change.

We use cross-sectional time-series data for LDCs from 1960 to 2010 to construct generalized least-squares panel regression models that assess the differing effects of decline and growth in urbanization (as measured by the concentration of populations in urban areas) on CO_2_ emissions. The goal of this study is to assess whether growth and decline in urbanization mitigate or increase CO_2_ emissions and whether growth has an asymmetric effect relative to decline.

## Literature review

Urbanization fundamentally alters the environment due to local development and its connection with wider production, consumption, trade, and transportations patterns. In order to sustain urban expansion, communities use environmentally hazardous technologies to increase the extraction rate of natural resources, which disrupts ecosystems and degrades the environment [10, 11]. Additionally, urban regions are heavily reliant on external energy sources to sustain themselves, which are mostly derived from fossil fuels [12, 13], making urban expansion a key contributor to climate change through the emission of CO_2_ [4].

Numerous empirical assessments of the relationship between urbanization and energy consumption (one of the main ways urbanization contributes to CO_2_ emissions) have found that urbanization increases energy use [14, 15, 16] as well as CO_2_ emissions [17, 18, 19]. However, recent analyses have found that the relationship between urbanization and CO_2_ emissions is not monolithic and varies based on region and type of urban development [20, 21, 22, 23, 24, 25, 26]. Overall, these studies suggest that the relationship between urbanization and CO_2_ emissions in developing countries, to a large extent, is different from in developed countries.

One such difference, which is a major reason that our focus in this analysis is solely on LDCs, is that urbanization in developing countries is not always an engine of industrial development and the expansion of production [27, 28, 29]. Therefore, urbanization in LDCs is not always heavily reliant on fossil fuel energy, at least not in the short term. Perhaps the best example of this is the urbanization that has occurred across much of the continent of Africa, which has led to extreme poverty connected with the creation of slums [30, 31]. Slums lack adequate access to electricity and other forms of energy tied to fossil fuel consumption. As a result, urbanization in LDCs is not as closely linked with CO_2_ emissions as it is in more developed regions. Furthermore, while historically rural-urban migration in developed nations was driven by the proliferation of fossil fuel use stemming from industrialization, in LDCs, rural-urban migration is often sparked by failed agricultural policies and regional conflict [27, 28, 29]. This makes urban development in LDCs less bound to fossil fuel use. Hence, since in recent decades urbanization has been most rapid in LDCs and we have theoretical reasons to expect urbanization and deurbanization processes to be different in LDCs than in more affluent nations, we focus our analysis on LDCs.

Although the overall trajectory of urbanization over the past several decades has been growth, for a variety of reasons, many nations have experienced periods of deurbanization. Perhaps the most vivid example of this is the collapse of the Soviet Union in the 1990s. The collapse of the Soviet Union was followed by a period of transition, where many former Soviet Republics began to shift into market economies [32, 33, 34, 35, 36]. During this period, former Soviet Republics moved away from centralized industrial economies to more household oriented forms of production, which were not connected to formal markets [37]. The economic decline in these countries was also associated with modest deurbanization, as measured by the proportion of the population that resided in urban areas [38]. Declines in development in these regions due to economic collapses resulted in a modest reduction of CO_2_ emissions relative to the general effect of increases of economic growth on CO_2_ emissions [38]. This is likely due to the presence of durable infrastructure (e.g., roads, rail lines) that remains even during times of economic contraction. In addition to former Soviet republics, there are a number of LDCs that have experienced deurbanization in between periods of urban growth. For example, in the late 1960s and early 70s during the Cultural Revolution, China experienced a decline in the urbanization of its population.

Periods of degrowth are also tied to factors that are unique to LDCs, such as those connected with the historical legacy of colonialism. For example, in Malaysia there has been a period of deurbanization due to the depletion of oil and gas reserves that were historically tied to its rapid urbanization [9]. Additionally, many individuals residing in urban areas in LDCs view their occupancy as temporary and maintain social ties with rural areas that, unlike in developed nations, are far less reliant on fossil fuels [39]. As a result, during periods of civil conflict (e.g. wars and political regime changes), individuals often move back to rural areas and resort to a way of life that is disconnected from fossil fuel use.

We assess the effects of deurbanization in LDCs and compare it to the effects of urbanization. Thus, we investigate the a/symmetry of deurbanization/urbanization to better understand the environmental implications of urban development. Our approach is similar to York’s [40] analysis of the a/symmetry of economic development’s connection to CO_2_ emissions, where it was found that the effect of declines in GDP per capita on CO_2_ emissions per capita was not symmetrical with the effect of increases in GDP per capita on CO_2_ emissions per capita. Specifically, the effect of declines in GDP per capita on CO_2_ emissions was smaller than the effect of growth on CO_2_ emissions. York [38, 40] argues that this asymmetry with respect to economic growth may occur because of “infrastructural momentum,” where durable goods, such as transportation networks, housing and sewage systems, created during periods of economic development stay in place during periods of economic declines, necessitating ongoing energy use. In a similar vein, here we ask if infrastructural momentum affects the relationship between urbanization/deurbanization and CO_2_ emissions. However, there are reasons to expect, counter to the infrastructural momentum pattern that characterizes the relationship between economic development and emissions, that deurbanization may have a larger effect than urbanization in LDCs, for the reasons we explain above. Since in LDCs energy intensive technologies (e.g., electricity, transit systems) are typically concentrated in cities and less available in rural areas, a shift of the population out of cities may lead to a sharp drop in energy consumption, and, therefore, CO_2_ emissions. In contrast, growth in the urban population may not have a large immediate effect on emissions, since new migrants often take time to integrate into the urban economy and establish an urban, energy intensive lifestyle. However, since the consequences of changes in urban population are surely complex, the exact consequences of de/urbanization are unclear. Our assessment here, therefore, is exploratory to determine the empirical pattern that has existed in LDCs in recent decades.

## Data and methods

We estimate a generalized least-squares panel model with robust standard errors adjusted for clustering of residuals by nation, which uses nation-years as its unit of analysis. All variables used in our models were obtained through World Bank’s [41] World Development Indicators. We include in our analysis the 102 nations (see Table A in S2 File) with populations greater than 500,000 identified by the World Bank as low or middle income for which data are available for all of the variables included in the model for at least some years from 1960–2010. We conducted exploratory models using developed nations, but we did not find a consistent pattern of asymmetry in these nations. The relationship we find appears to be particular to less developed nations. We believe this is for the theoretical reasons we explain above. In particular, urban to rural migration in developed nations may not result in substantial reductions in fossil fuel use as they do in less developed countries since rural areas in developed countries often contain infrastructure that relies on fossil fuels such as, electrical grids, and roads for vehicle transportation. Some of the years are excluded for some nations due to missing data, most commonly during the early periods of the time examined. We include dummy variables for each year to account for general period effects. All variables are in natural logarithmic form, making this an elasticity model. All variables included in the analyses were first differenced (after logging), which makes their values representative of the annual change in the variable under consideration. Thus, we are using the first-difference estimator, which is similar to using a fixed-effects model (hence why we do not use the fixed-effects estimator), in that it controls for factors that vary across nations, but are temporally invariant (such as geography) [42]. Additionally, first-differencing is needed to allow us to examine directional asymmetry by identifying growth as separate from decline in urbanization [7]. This is the same approach used by York [40] to assess the asymmetry of economic development and CO_2_ emissions.

Our dependent variable is metric tons of CO_2_ emissions from fossil-fuel combustion and cement manufacturing per capita (see the World Bank [41] for a full description of how this and the other variables are measured). Our primary independent variable of interest is the percent of individuals in a nation living in an urban area (as defined by national statistical offices). Although there are other ways to measure urbanization that have been used by other researchers, such as prevalence of slums [43] and amount of impervious surface area [21], these alternative measures have poor data coverage across nations and over time. Hence, we focus on the commonly used measure of population concentration in urban areas. We also control for GDP per capita (in constant 2010 US dollars), percentage of GDP coming from manufacturing sector, the proportion of people between 15 years of age and 64 years of age, and exports of goods and services as a percentage of GDP.

The logic of our modeling approach is to control for time-variant factors that are known to influence CO_2_ emissions at the national level and assess the effect of increases and decreases in urbanization separately in one model. To accomplish this, we created separate independent variables for increases and decreases in urbanization, where the increase variable is coded as 0 if there are decreases and the decrease variable is coded as 0 if there are increases, but otherwise the variables have their observed values [7, 40]. Our goal is to determine whether the effect size of increases and decreases in urbanization are significantly different from zero and from each other, which we assess with a post-test.

Specifically, the model we estimate (using the “xtreg” command with the “robust” option in STATA 14) is

$$\Delta \text{ln}\left({\text{CO}}_{2}\text{per}{\text{capita}}_{\text{it}}\right)=\text{a}+{\text{b}}_{1}(\Delta \text{ln}(\text{urban}{\text{decrease}}_{\text{it}}))+{\text{b}}_{2}\left(\Delta \text{ln}\left(\text{urban}{\text{increase}}_{\text{it}}\right)\right)+{\text{b}}_{3}(\Delta \text{ln}(\text{GDP}\;\text{per}{\text{capita}}_{\text{it}}))+{\text{b}}_{4}(\Delta \text{ln}({\text{manufacturing}}_{\text{it}}\text{)}+{\text{b}}_{5}\left(\Delta \text{ln}\left(\text{population}\;\text{age}15-64_{\text{it}}\right)\right)+{\text{b}}_{6}(\Delta \text{ln}({\text{exports}}_{\text{it}}))+{\text{t}}_{\text{t}}+{\text{e}}_{\text{it}}$$

where a is the y-intercept (the predicted annual change in the dependent variable if all independent variables remain constant), t_t_ is the period effect common across nations for each point in time (measured by dummy-variables for each year), e_it_ is the disturbance term unique to each nation at each point in time, and “ln” indicates the natural logarithm.

## Results

The results of our principal analysis are reported in Table 1. In the asymmetric model, both the percentage of population age 15–64, exports as a percentage of GDP, and the percentage of GDP from manufacturing did not have a significant effect on CO_2_ emissions. GDP per capita had a positive effect on CO_2_ emissions, which is consistent with previous findings in similar analyses [3, 44]. In a model discussed below, we assessed whether GDP per capita had significant asymmetry in its effect, as York [40] found. In our model, the effect was not significantly asymmetrical, likely due to the limited sample of nations we focus on here (LDCs). Therefore, in the principal model we present, we do not separate increases from decreases in GDP per capita.

**Table 1:** Principal models. Generalized least-squares panel regression models of the influence on CO_2_ per capita in LDCs, 1960–2010. All variables are in natural logarithmic form. All models include year dummy variables (not shown) to control for period effects. The standard errors are robust, accounting for clustering by nation.

Independent variables	Model 1 Asymmetric urbanization Coefficients (standard errors)	Model 2 Symmetric urbanization Coefficients (standard errors)
**Urbanization (% of population) increase**	.655* (.295)	
**Urbanization (% of population) decrease**	5.332** (1.758)	
**Urbanization (%)**		.783** (.295)
**GDP per capita**	.466*** (.107)	.479*** (.108)
**Manufacturing (% of GDP)**	**.011** **(.042)**	**.012** **(.042)**
**% of population age 15–64**	**-.528** **(.726)**	**-.508** **(.717)**
**Exports as a percentage of GDP**	**.007** **(.026**	**.007** **(026)**
**R**^**2**^ **(within)**	**.060**	**.058**
**N total/nations**	**102/2,009**	**102/2,009**

*** p<.001

** p<.01

* p<.05

† p<.10

(two-tailed tests)

The main variables of interest in our model (Model 1), urbanization increase and urbanization decrease, test for asymmetry in the effect that change in urbanization has on CO_2_ emissions per capita. Note that because our model is an elasticity model, it already allows for a nonlinear relationship between CO_2_ emissions and urbanization. Furthermore, in a model discussed below, we further assessed the potential of a nonlinear relationship between urbanization and CO_2_ emissions by including a quadratic term for both urban increase and urban decrease. However, the quadratic term was not found to be significant at a .05 level (two-tailed test) for either variable. Findings suggest that the effect of increases and decreases in urbanization on CO_2_ emissions is asymmetrical. Specifically, a 1 percent increase in urbanization in LDCs is associated with approximately a .66 percent increase in CO_2_ emissions per capita, while a 1 percent decrease in urbanization is associated with approximately 5.33 percent decrease in CO_2_ emissions. A post-test established that the coefficients for increase and decrease are significantly different from one another at the .05 level (two-tailed test). Note that because our urbanization variable is the natural log of a percent measurement, our coefficients for urbanization increase and decrease represent the effect of a 1% percent change in the proportion of the population living in urban areas on CO_2_ emissions.

**Fig 1:**
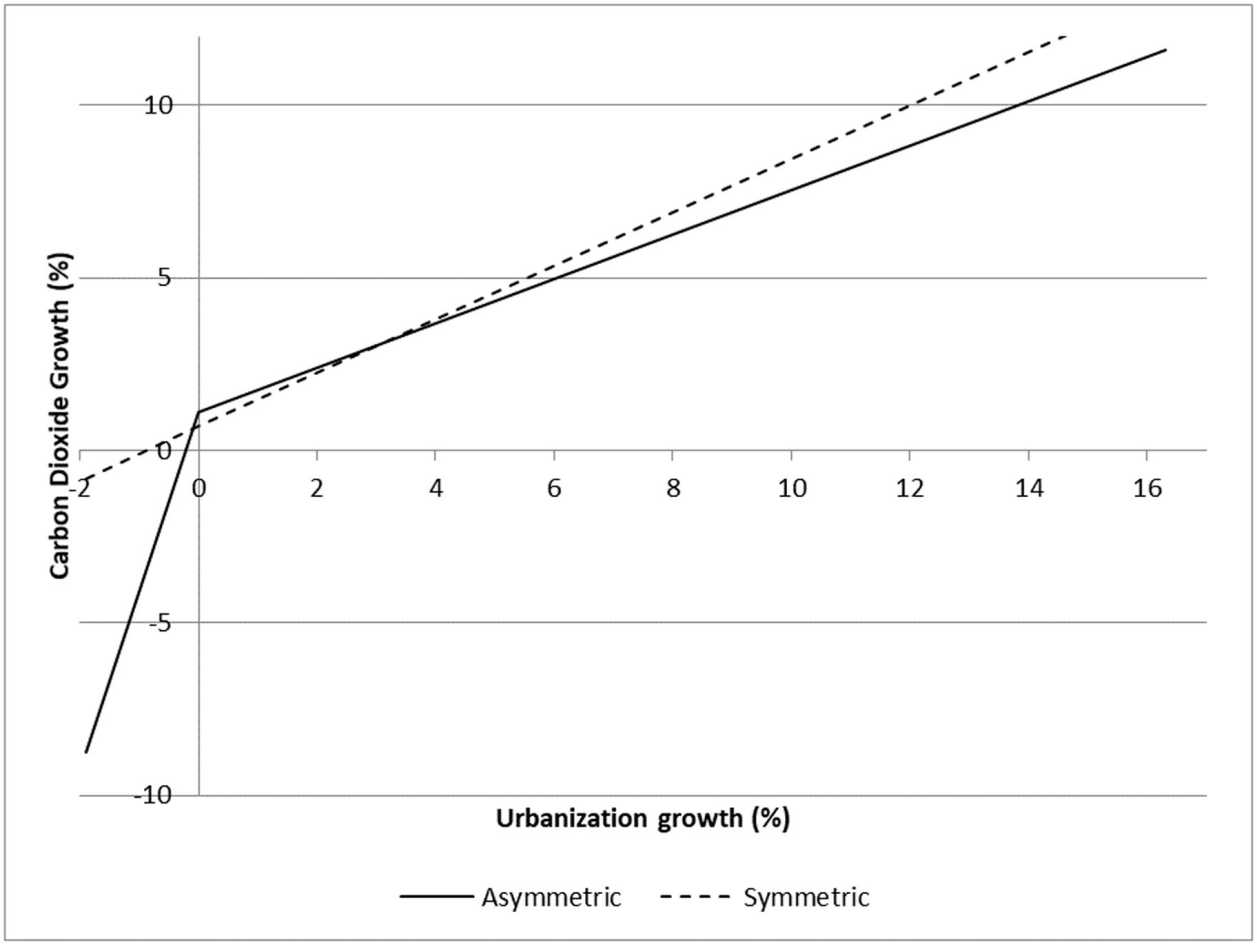
Effect of change in urbanization on CO_2_ emissions from asymmetric and symmetric models. Calculated based on the urbanization coefficients presented in Table 1 with the y-intercept calculated based on averaging all of the year-dummies.

In Table 1, we also present a model (Model 2) of CO_2_ emissions that does not allow for an asymmetric effect from urbanization, like the typical model in the literature. To show the distinction between the estimated relationship when asymmetry is taken into account and when it is not, we present in Fig 1 the estimated effect (based on the regression coefficients presented in Table 1) of change in urbanization on CO_2_ emissions for both the asymmetric and symmetric models (Models 1 and 2). In order to compare symmetrical and asymmetrical estimates, we need a single y-intercept for each model (since the intercept varies from year to year due to the year-dummies), so we used the mean of the year-dummies across all periods. Using any single year for the intercept will not change the slopes of the lines, but will shift the lines up or down the y-axis. In Fig 1, the range of values for change of urbanization is constrained to the range of observations included in our model.

As can be seen in Fig 1, the symmetrical model underestimates the contribution of urbanization to growth in emissions when the annual rate of change is between approximately 0% and 3.4% and overestimates it outside of this range. Additionally, as can be seen by the range of change in urbanization in the figure, when deurbanization occurs, it is typically at a low rate. There are no annual declines in urbanization more substantial than -2 percent. By comparison, in some nations at some times, increases in urbanization have been much larger, with the largest annual increase reaching over 16 percent. Thus, while our model estimates that, for equal amounts of change, declines in urbanization substantially decrease CO_2_ emissions compared to the effect of growth, instances of deurbanization are relatively rare (about 10 percent of observations) and not very large. In contrast, growth in urbanization is the norm, and sometimes the rate of growth is large. Therefore, even though the effect of each increment of growth on emissions is modest, the fact that growth is common and substantial means it drives up emissions cumulatively over time. Nonetheless the effect of deurbanization is pronounced when it occurs and is not symmetrical with the effect of growth in urbanization.

We have estimated a number of auxiliary models to assess whether our key finding (asymmetry in de/urbanization) holds, which we present in Table 2. In Model 3, we present the results from the model mentioned above assessing asymmetry in the effect of GDP per capita as well as urbanization. There is no significant asymmetry in GDP per capita, and the results for urbanization are consistent with those presented in Model 1. In Model 4 we present the results from the model mentioned above assessing whether urbanization has a quadratic relationship with emissions. Neither of the quadratic terms (for increase or for decrease) are significant, indicating that the specification in Model 1 is appropriate. In Model 5, we exclude former Soviet republics to assess whether our results are driven by those nations. The results are similar to those presented in Model 1. However, the level of significance of the post-test for the difference between the urbanization increase and urbanization decrease coefficients is only at the .10 level (two-tailed test). This is not surprising since excluding the former Soviet republics reduces sample size. In Model 6, we examine only the time period from 1960–1985 to assess whether our findings hold across the full range of observations or are only particular to part of the period examined. The findings are consistent with those presented in Model 1, although the significance level for the post-test of the difference between urbanization increase and urbanization decrease is only at the .10 level (two-tailed test). The lower level of significance is not surprising given the much smaller sample size. In Model 7 we analyzed only the time period from 1985–2010. The results are very similar to those presented in Model 1, and the difference between urbanization increase and urbanization decrease remains significant at the .05 level (two-tailed test). These models taken together suggest that the asymmetry between urbanization and de-urbanization is common across time and not driven by changes in former Soviet republics.

**Table 2:** Auxiliary models. Generalized least-squares panel regression models of the influence on CO_2_ per capita in LDCs. All variables are in natural logarithmic form. All models include year dummy variables (not shown) to control for period effects. The standard errors are robust, accounting for clustering by nation.

Independent variables	Model 3 Asymmetric GDP p.c. Coef. (S.E.)	Model 4 Quadratic urban. Coef. (S.E.)	Model 5 No Former Soviets Coef. (S.E.)	Model 6 Only 1960–1985 Coef. (S.E.)	Model 7 Only 1985–2010 Coef. (S.E.)
**Urbanization (% of population) increase**	.662* (.312)	**1.032**† **(.550)**	.661* (.307)	**.453** **(.331)**	**.776**† **(.420)**
**Urbanization (% of population) decrease**	5.261** (1.765)	**3.449** **(3.296)**	4.250* (2.019)	7.331* (3.885)	5.352* (2.143)
**Quadratic of urbanization (%) increase**		**-4.075** **(3.410)**			
**Quadratic of urbanization (%) decrease**		**-131.527** **(186.064)**			
**GDP per capita**		.461*** (.107)	.409*** (.115)	.574* (.166)	.420** (.128)
**GDP per capita increase**	.449** (.168)				
**GDP per capita decrease**	.485** (.151)				
**Manufacturing (% of GDP)**	**.011** **(.042)**	**.010** **(.042)**	**-.010** **(.046)**	.173* (.086)	**-.030** **(.047)**
**% of population age 15–64**	**-.517** **(.724)**	**-.455** **(.729)**	**-.804** **(.772)**	**-1.023** **(1.127)**	**-.314** **(.896)**
**Exports as a percentage of GDP**	**.007** **(.026)**	**.007** **(.026)**	**.003** **(.027)**	**-.054** **(.043)**	**.034** **(.031)**
**R**^**2**^ **(within)**	**.060**	**.060**	**.053**	**.103**	**.043**
**N total/nations**	**2009/102**	**2009/102**	**1905/91**	**650/57**	**1359/101**

*** p<.001

** p<.01

* p<.05

^†^ p<.10

(two-tailed tests)

It is important to note, however, that our findings should not be generalized beyond the nations and time periods included in our analysis. Since there are only a limited number of instances of deurbanization in these nations, the estimated effect of deurbanization is liable to be affected by the addition or the subtraction of some nations. A different definition of which nations are LDCs and which not would lead to a different group of nations, for which results may be different from those we present here. Additionally, there are a number of nations that are excluded from our analysis due to data limitations, and it is entirely possible that processes operate differently in those nations. Thus, changes in the sample (which could occur if other variables were added to the model that have more limited coverage or if variables which are missing for some nations are removed from the model) may lead to different results. This is situation is not different from other analyses of non-experimental data, but it is important to take into consideration.

## Discussion and conclusion

The present study shows that in LDCs examined here urbanization is a key factor driving the expansion of CO_2_ emissions, consistent with the previous findings on the effect of urbanization on CO_2_ emissions. We also found an important subtlety in the relationship between urbanization and CO_2_ emissions. The effect of growth in urbanization on CO_2_ emissions is not symmetrical with the effect of decline in urbanization. Specifically, for equal amounts of change in urbanization, declines in urbanization lead to substantially bigger decreases in emissions than urban growth leads to increases. However, growth in urbanization is much more common than deurbanization, and when deurbanization occurs it is typically only to a modest degree. This finding suggests that there is something distinctive about deurbanization, and that it is not a simple reverse of urbanization. One explanation is that deurbanization may be connected with various processes of destabilization (e.g., war, political unrest) which may disrupt electricity production, transportation, and other energy dependent processes. Another explanation is related to circular migration patterns. In LDCs, urban migrants often maintain connections to rural areas and eventually return to these regions creating a cycle of rural to urban and urban to rural domestic migration. While circular migration is a common pattern in LDCs, various factors contribute to net increases in urban-rural migration, such as industrial decline and increases in urban poverty. As a result, deurbanization reduces CO_2_ emissions produced from industry as well as CO_2_ emissions that derive from urban living (e.g. vehicle use, electricity consumption etc.), but urbanization contributes less to emissions in the short-term since new migrants take time to integrate into the urban economy.

We find the opposite of what may be expected from the process of “infrastructural momentum” that has been found to characterize the relationship between economic development and emissions. York [40] noted that when economies grow, they build infrastructure, such as roads and power plants, which promotes energy consumption and is durable, remaining during economic downturns. Therefore, energy consumption and CO_2_ emissions may not decline as much when the economy shrinks as they grow when the economy grows [35, 39]. It could be the case that a similar process may occur with urbanization, but we found the opposite, where deurbanization leads to a greater decline in emissions than urbanization leads to growth in emissions. It is important to note that York’s [40] analysis included a wider sample of nations, and the processes we are examining likely differ across nations and national groupings. Nonetheless, a potential explanation for our results is that energy intensive processes, such as electricity and fossil fuel powered transit systems, are concentrated in cities, especially in LDCs, so that a movement of population away from cities reduces the potential for energy consumption. Unlike economic growth, urbanization measures individuals’ spatial distribution and connections to land. Thus, the concept of infrastructural momentum, devised by York, needs further refinement regarding how it applies and in what contexts.

Our finding points to two likely scenarios. 1.) It may be the case the rural-urban migration operates differently in LDCs compared to in developed countries due to the lack of fossil fuel based infrastructure in many LDCs. Case studies of deurbanization have found that urban inhabitants in LDCs have a unique relationship to land that is embedded in ancestral legacies, suggesting that rural inhabitants in LDCs are less reliant on fossil fuels [39]. 2.) Declines in economic growth may not always spark deurbanization. Individuals may stay in urban areas during economic downturns, resulting in infrastructural momentum with respect to economic growth if not urbanization. This suggests that deurbanization may be a path toward reduced emissions. Many LDCs, such as Malaysia, already have policies that facilitate urban-rural migration [9], which may be a factor in our findings. In sum, while the mechanism that spark deurbanization may be diffuse and difficult to pin point, the outcome of deurbanization is often migration to rural areas that use less fossil fuels. In these instances, it seems likely that deurbanization would result in substantially lower CO_2_ emissions. However, additional research is necessary to fully understand this process.

As York and Light [7] note, a finding of asymmetry is not so much an explanation of a phenomenon, as the identification of a phenomenon that needs to be explained. We present this result in that spirit, and recognize the need for further research to sort out the processes that are responsible for this asymmetry. Further analyses are necessary to establish how general our findings are; in particular to determine the extent to which asymmetry is found in other groupings of nations, across various temporal periods. Our findings may result from a complex, multi-causal, and multi-directional process connecting urbanization and emissions with other factors. Quantitative analyses that take additional factors into account may help to identify some of the forces underlying these results. Perhaps more importantly, historical analyses of nations when they experienced declines in proportion of the population living in urban areas may give insight into the processes occurring when deurbanization happens.

Deurbanization in LDCs, although rare, is not simply an undoing of urbanization and should be understood in its own context. In our data, deurbanization occurs in multiple nations and in a variety of years, (see Table B in S2 File) making it difficult to ascertain a definitive link between the causes of deurbanization and the effect of deurbanization on the environment. Moreover, future research exploring the relationship between urbanization and CO_2_ emissions should assess the temporal patterns of urban development as well as the absolute level of urbanization on CO_2_ emissions. Understanding why deurbanization reduces CO_2_ emissions more than urbanization increases CO_2_ emissions in the LDCs we examine here may require a further convergence of theoretical perspectives in environmental sociology, sociology of development, and urban planning.

## Supporting information

S1 File Raw data. This file contains all data used to perform the analyses presented by the authors above.
(CSV)

S2 File All nations included in the models **(Table A)**. List of Countries and years when the percent of urban population declined. Note that years included in this table indicate end years (e.g., 2005 means a period where there was decline from 2004–2005) **(Table B)**.
(DOCX)
