# Supplementary material for: Asymmetric relationship of urbanization and CO2 emissions in less developed countries
Source: PLoS One. 2018 Dec 7;13(12):e0208388. doi: 10.1371/journal.pone.0208388 (PMC6286174; doi:10.1371/journal.pone.0208388)
Supplement: S2 File — All nations included in the models (Table A). List of Countries and years when the percent of urban population declined. Note that years included in this table indicate end years (e.g., 2005 means a period where there was decline from 2004–2005) (Table B). (DOCX) [file pone.0208388.s002.docx]

**S1 Table1**: All nations included in the models.

Afghanistan, Algeria, Angola, Argentina, Armenia, Azerbaijan, Bangladesh, Belarus, Benin, Bhutan, Bolivia, Botswana, Brazil, Burkina Faso, Burundi, Cambodia, Cameroon, Central African Republic, Chad, China, Columbia, Comoros, Congo Democratic Republic, Congo Republic, Costa Rica, Cote d’Ivoire, Cuba, Djibouti, Dominican Republic, Ecuador, Egypt, El Salvador, Eritrea, Ethiopia, Fiji, Gabon, Gambia, Georgia, Ghana, Guatemala, Guinea, Guinea-Bissau, Guyana, Honduras, India, Indonesia, Iran, Jamaica, Jordan, Kazakhstan, Kenya, Kyrgyz Republic, Lao People’s Democratic Republic, Lebanon, Lesotho, Libya, Macedonia, Madagascar, Malawi, Malaysia, Mali, Mauritania, Mauritius, Mexico, Moldova, Mongolia, Montenegro, Morocco, Mozambique, Namibia, Nepal, Nicaragua, Niger, Nigeria, Pakistan, Panama, Papua New Guinea, Paraguay, Peru, Philippines, Romania, Russia, Rwanda, Senegal, Serbia, Sierra Leone, South Africa, Sri Lanka, Sudan, Suriname, Swaziland, Tajikistan, Tanzania, Thailand, Timor-Leste, Togo, Tunisia, Turkey, Turkmenistan, Uganda, Ukraine

**S2 Table1**: List of Countries and years when the percent of urban population declined. Note that years included in this table indicate end years (e.g., 2005 means a period where there was decline from 2004-2005).

| **Country** | **Years** |
| --- | --- |
| **Armenia** | **1993, 1995, 1996, 1997, 2000** |
| **Azerbaijan** | **1997, 1998, 1999** |
| **China** | **1965, 1967, 1968, 1970, 1971** |
| **Comoros** | **1998, 1999, 2000, 2001, 2002, 2003, 2006** |
| **Egypt** | **1987, 1988, 1991, 1993,1996** |
| **Georgia** | **1997, 1998, 1999, 2000, 2001, 2002** |
| **Guyana** | **1982, 1986, 1987, 1988, 1991, 1993, 1995, 1996, 2000, 2004, 2006, 2007, 2008** |
| **Kazakhstan** | **2000, 2001, 2002, 2003, 2004, 2006 2009, 2010** |
| **Kyrgyz Republic** | **1993, 1996, 1998, 2001, 2003, 2005, 2008, 2009** |
| **Macedonia** | **1995, 1996, 1997, 1998 1999, 2001, 2003, 2005, 2006, 2007, 2008, 2009** |
| **Mauritius** | **1977, 1979, 1980, 1981, 1982, 1983, 1991, 1997, 1998, 2000, 2001, 2004, 2006, 2007, 2008, 2009, 2010** |
| **Moldova** | **1995, 1997, 1998, 1999, 2000, 2001, 2002, 2003, 2004, 2005, 2006, 2007, 2008, 2010** |
| **Mongolia** | **1990, 1991, 1992, 1993, 1994, 1995, 1996, 1997, 1998, 1999** |
| **Papua New Guinea** | **1991, 1993, 1994, 1995, 1996, 1997, 1998, 2000, 2001, 2003, 2004** |
| **Philippines** | **1991, 1992, 1993, 1994, 1995, 1996, 1998, 1999, 2000, 2001, 2002, 2003, 2004, 2005, 2006, 2007, 2010** |
| **Romania** | **1993, 1994, 1996, 1998, 1999, 2000, 2001** |
| **Sri Lanka** | **1973, 1974, 1975, 1976, 1977, 1979, 1980, 1982, 1983, 1984, 1986, 1988, 1989, 1990, 1991, 1992, 1993, 1994, 1997, 1998, 2000, 2001, 2002, 2003, 2004, 2006, 2009, 2010** |
| **Suriname** | **2007, 2008, 2009, 2010** |
| **Swaziland** | **1998, 1999, 2000, 2001, 2003, 2004, 2005, 2006, 2007, 2008, 2009** |
| **Tajikistan** | **1993, 1994, 1995, 1996, 1997, 1998, 2000, 2001, 2003** |
| **Turkmenistan** | **1994** |
